# Influence of Maternal Supplementation with Vitamins, Minerals, and (or) Protein/Energy on Placental Development and Angiogenic Factors in Beef Heifers during Pregnancy

**DOI:** 10.3390/vetsci11030111

**Published:** 2024-03-02

**Authors:** Bethania J. Dávila Ruiz, Carl R. Dahlen, Kacie L. McCarthy, Joel S. Caton, Jennifer L. Hurlbert, Friederike Baumgaertner, Ana Clara B. Menezes, Wellison J. S. Diniz, Sarah R. Underdahl, James D. Kirsch, Kevin K. Sedivec, Kerri A. Bochantin, Pawel P. Borowicz, Sebastián Canovas, Lawrence P. Reynolds

**Affiliations:** 1 Center for Nutrition and Pregnancy, and Department of Animal Sciences, North Dakota State University, Fargo, ND 58108, USA; bethania.davilaruiz@ndsu.edu (B.J.D.R.); carl.dahlen@ndsu.edu (C.R.D.); joel.caton@ndsu.edu (J.S.C.); jennifer.hurlbert@ndsu.edu (J.L.H.); riederike.baumgrtne@ndsu.edu (F.B.); sarah.underdahl@ndsu.edu (S.R.U.); james.kirsch@ndsu.edu (J.D.K.); pawel.borowicz@ndsu.edu (P.P.B.); 2 Department of Animal Sciences, University of Nebraska-Lincoln, Lincoln, NE 68583, USA; kacie.mccarthy@unl.edu; 3 Department of Animal Sciences, South Dakota State University, Brookings, SD 57006, USA; anaclara.baiaomenezes@sdstate.edu; 4 Department of Animal Sciences, Auburn University, Auburn, AL 36832, USA; wzd0027@auburn.edu; 5 Central Grasslands Research Extension Center, North Dakota State University, Streeter, ND 58483, USA; kevin.sedivec@ndsu.edu; 6 Physiology of Reproduction Group, Physiology Department, Mare Nostrum Campus, University of Murcia, 30100 Murcia, Spain; scber@um.es

**Keywords:** angiogenic factors, cotyledons, early pregnancy, gene expression, mineral nutrition, placental vascularity, vitamins

## Abstract

**Simple Summary:**

Understanding placental vascularity is vital for ensuring the proper nourishment of the fetus and, therefore, a healthy offspring. We aimed to investigate the impact of vitamin and mineral supplementation and/or different rates of body weight gain on placental vascularity in beef heifers. To this end, in the first experiment, heifers were divided into groups that received vitamin and mineral supplementation or did not at least 72 days before breeding. At breeding, they were further divided into low or moderate-weight gain groups, resulting in four different treatments maintained until day 83 of pregnancy when tissue collection was performed. In the second experiment, another group of heifers received a basal diet or a diet with vitamin and mineral supplementation from breeding until parturition. We evaluated placental blood vessel density in both experiments and the placental expression of genes related to blood vessel growth in the first experiment. Results showed that supplementation and the rate of body weight gain during early pregnancy did not significantly affect placental vascularity or the expression of angiogenic factors. On the other hand, placental vascularity measured at parturition was increased in the fetal placenta of the supplemented group. These findings suggest that supplementation with vitamins and minerals throughout all gestation may impact placental function at a later stage of pregnancy.

**Abstract:**

The effect of vitamins and minerals supplementation (VTM) and/or two rates of body weight gain (GAIN) on bovine placental vascular development and angiogenic factors gene expression were evaluated in two experiments: In Exp. 1, crossbred Angus heifers (*n* = 34) were assigned to VTM/NoVTM treatments at least 71 days before breeding to allow changes in the mineral status. At breeding, through artificial insemination (AI), heifers were assigned to low-gain (LG) 0.28 kg/d or moderate-gain (MG) 0.79 kg/d treatments, resulting in NoVTM-LG (Control; *n* = 8), NoVTM-MG (*n* = 8), VTM-LG (*n* = 9), and VTM-MG (*n* = 9) until day 83 of gestation; In Exp. 2, crossbred angus heifers (*n* = 28), were assigned to control (CON; *n* = 12), receiving a basal total mixed ration (TMR) or TMR + VTM (VTM; *n* = 16) from breeding until parturition. Placentomes from Exp. 1 and cotyledons (COT) from Exp. 2 were evaluated by immunohistochemistry for COT vascular density area. COTs from Exp. 1 were evaluated for angiogenic factor (*ANGPT*-1, *ANGPT*-2, *eNOS*2, *eNOS*3, *FLT*1, *KDR*, *TEK*, *VEGFA*) gene expression. In Exp. 1, COT vascularity was not affected by the interaction of VTM and GAIN (*p* = 0.67) or the main effects of VTM (*p* = 0.50) and GAIN (*p* = 0.55). Likewise, angiogenic factors were not differentially expressed between treatments (*p* < 0.05). In Exp. 2, COT vascularity was greater in VTM vs. CON (*p* = 0.07). In conclusion, there is a suggested later-stage influence of vitamin and mineral supplementation on placental vascularity, emphasizing the importance of supplementation beyond early pregnancy.

## 1. Introduction

One of the earliest processes during embryonic development is the establishment of a functional fetoplacental vascular bed [1,2]. In cattle, during the first 50 days of pregnancy, the fetoplacental vascular bed is formed [3]. Nutrients and oxygen diffuse from the maternal blood into the fetoplacental villi and then into the fetal blood and, conversely, with carbon dioxide and metabolic wastes. Therefore, when fetoplacental circulation is established, the source of nutrition to the fetus changes from histotrophic to hemotrophic over time [1,2,4]. Placental vascular growth enables placental transport to keep up with the demands of the conceptus for oxygen, nutrients, and metabolic substrates as it grows; thus, the formation of a highly branched vascular network is a critical determinant for placental function [3,5,6]. Conversely, reduced uteroplacental vascular development is a major cause of fetal growth restriction and low birth weight, which are major determinants of postnatal survival, growth, and health of the offspring [7,8].

Previous studies have demonstrated that in pregnancies that are compromised from factors, such as an increased number of fetuses, maternal age, assisted reproductive techniques, or maternal nutritional stress, placental angiogenesis is abnormal at term [5,9,10,11,12]. Therefore, poor placental vascular development in the early stages of pregnancy can predict high-risk pregnancies by increasing uteroplacental vascular resistance and reducing uterine blood flow [3,10]. Placental growth occurs primarily during the first half of gestation, with some variations between ruminant species [1]. In cattle, placental weight increases exponentially throughout gestation, although it slows during the last half of pregnancy, while in sheep, after day 90 of gestation, placental weight either stops increasing or even slightly decreases [5]. However, despite these observations concerning placental growth, placental transport capacity increases with fetal growth because placental vascular development is continual, resulting in increased vascular density throughout gestation [3].

The *de novo* development of blood vessels (vasculogenesis) and the expansion and elongation of pre-existing blood vessels (angiogenesis) are responsible for the development of the new vascular beds during the embryonic and post-embryonic periods, respectively [1,2,3,4,6]. Both processes depend on angiogenic/anti-angiogenic factors, including placental growth factor (PlGF), vascular endothelial growth factor (VEGF), angiopoietin-1 (Ang-1), angiopoietin-2 (Ang-2), soluble fms-like tyrosine kinase-1 (sFlt-1), soluble endoglin (sEng), and nitric oxide (NO) [3,7,13,14]. While not typically considered primary angiogenic factors, eNOS2 and eNOS3, which generate the potent angiogenic factor NO from arginine, play roles in angiogenesis. It is important to keep in mind that these factors are also being secreted into the maternal circulation, and in conjunction with hormones, act to enable adequate maternal adaptation to pregnancy, including increased cardiac output and blood volume, while at the same time inducing vasodilatation of resistance vessels to avoid high maternal blood pressure [3,7,13].

One of the primary influences on developmental programming in offspring is maternal nutrition [5,15,16]. During pregnancy, the dam’s nutrient demand increases to meet the growing needs of the conceptus, further compounded by the ongoing growth demands of the heifers themselves [17]. Therefore, using a heifer as a model will have a high sensitivity to maternal nutritional perturbations. Macro (energy, protein, and fats) and micronutrients (vitamins and minerals, VTM) are essential to developing a successful pregnancy [16,17]. The impact of micronutrients on placental function has been extensively studied due to the prevalence of micronutrient deficiencies in pregnancy, attributed to the increased demand for nutrients [18,19]. Specific placental nutrient transporters, such as those for vitamins B6, B12, C, folate, iron, and zinc, may be upregulated in pregnancies complicated by micronutrient insufficiency to sustain fetal supply. However, this response is not uniform across all micronutrients [20].

Micronutrient status has been linked to molecular mechanisms controlling placental vascularization [18,19]. For instance, elevated Vitamin E levels may enhance placental expression of VEGF [21]. Conversely, dietary restriction of Vitamin D in mice, both preconceptionally and during pregnancy, resulted in notable placental morphological changes, reduced placental weight, vascularization, and expression of VEGF [22]. Intriguingly, high-dose Vitamin D supplementation in pregnant rats also led to reduced placental size and abnormal vascularization [23]. Moreover, maternal selenium (Se) supplementation during pregnancy has impacted vascular development and the expression of COT angiogenic factors [24]. Furthermore, the maternal status of vitamins and minerals is proposed to involve several biological and biochemical processes, including placental growth, metabolism, transport, and morphology [25,26]. Additionally, it influences hormonal regulatory pathways [27], which is particularly relevant to placental vascular development, as estrogen plays a crucial role in regulating the expression of angiogenic factors [28]. Although growing evidence has shown the impact of macronutrients on fetoplacental development and function, micronutrients have not received the same attention [16], and no studies have investigated the effects of vitamin and mineral supplementation throughout gestation on placental vascular development in beef cattle to date.

Herein, we hypothesized that dietary vitamin and mineral supplementation (VTM) and/or different body weight gain (GAIN) rates throughout gestation would improve fetoplacental development and function in beef heifers. Our main objective was to evaluate the placental vascular density area and whether it is affected by VTM and/or GAIN supplementation. We evaluated how VTM and/or GAIN supplementation affects placental vascular development and angiogenic factor gene expression by collecting placental samples from beef heifers from the first third of gestation and at parturition.

## 2. Materials and Methods

### 2.1. Ethics Statement

All animal procedures were approved by the North Dakota State University Institutional Animal Care and Use Committee (IACUC A19012).

### 2.2. Animals and Experimental Design

Two different experiments were conducted as described below.

#### 2.2.1. Experiment 1: Maternal Supplementation from Pre-Breeding to Day 83 of Gestation

Details of the experiment design and outcomes were reported by Menezes et al. 2022 [29]. As a brief description, crossbred Angus heifers (*n* = 34; body weight = 359.5 ± 7.1 kg) were randomly assigned to one of four treatment groups. The treatments comprised a 2 × 2 factorial arrangement, with the main factors of vitamin and mineral supplementation (VTM) and two rates of body weight gain (GAIN). The VTM supplementation was implemented at least 71 days before artificial insemination (AI) to allow changes in the heifer’s mineral status before breeding. The two rates of gain (low gain (LG) 0.28 kg/d or moderate gain (MG) 0.79 kg/d) were assigned at the day of breeding. The factorial arrangement resulted in the following treatments:

1. No vitamin and mineral supplementation and low gain (Control; NoVTM-LG; *n* = 8);
2. No vitamin and mineral supplementation and moderate gain (NoVTM-MG; *n* = 8);
3. Vitamin and mineral supplementation but low gain (VTM-LG; *n* = 9); and
4. Vitamin and mineral supplementation and moderate gain (VTM-MG *n* = 9).

Diets were delivered once daily via a total mixed ration (TMR) consisting of corn silage, ground corn, triticale hay, and modified distiller’s grains for LG treatment, and the addition of the starch-based protein/energy supplementation consisting of a blend of ethoxyquin, urea, wheat midds, fish oil, ground corn, and a dried distillers’ grains plus solubles for MG treatment, plus the addition of vitamin and mineral mix if indicated. The VTM supplementation (113 g·heifer^−1^·d^−1^ of vitamin and mineral supplement [Purina Wind & Rain Storm All-Season 7.5 Complete, Land O’Lakes Inc., Arden Hills, MN, USA]) provided macro and trace minerals and vitamins A, D, and E to meet 110% of the requirements specified by the NASEM. The NoVTM supplement was a pelleted product feed (0.45 kg·heifer^−1^·day^−1^) with no added vitamin and mineral supplement. The specific nutrient composition of the total mixed ration and supplements provided is described in Table 1. As per the dosing precision, the specific amounts of the respective treatment feed ingredients were weighed daily for each heifer and placed in individual feed bunks equipped with an electronic feeding system (American Calan; Northwood, NH, USA) that only allowed access to one specified heifer to consume the feed.

Heifers were bred via AI using female-sexed semen from a single sire. Only heifers confirmed pregnant with female fetuses via transrectal ultrasound on day 65 after AI remained in the experiment. The heifers were maintained on their respective diets until day 83 ± 0.27 of gestation, when a hysterectomy was performed following the methodology outlined in McLean et al. 2016 [30], and uteroplacental tissues were collected. The largest placentome closest to the fetus was collected and processed for histological analysis as described below. Additionally, the maternal caruncle (CAR) and fetal cotyledon (COT) portions of the placenta were manually dissected.

#### 2.2.2. Experiment 2: Maternal Supplementation with Vitamins and Minerals from Breeding to Parturition

Details of the experimental design and resultant responses of the mineral status in the dam and offspring can be found in Hurlbert et al. 2024 [31]. Briefly, crossbred Angus heifers (*n* = 28) were randomly assigned to one of two treatment groups at breeding: either control (CON; *n* = 12) heifers receiving a basal diet consisting of a blend of corn silage, prairie hay, millet hay, alfalfa, DDGS, and a premix as in Exp. 1 without VTM supplement; or (VTM; *n* = 16) heifers receiving the basal diet with the addition of a VTM supplement (113 g·heifer^−1^·d^−1^ of a vitamin and mineral supplement [Purina Wind & Rain Storm All-Season 7.5 Complete, Land O’Lakes Inc., Arden Hills, MN, USA]). As per dosing precision, from the day of breeding (d 0) to day 106 of gestation, specific amounts of the respective treatment feed were weighed daily for each heifer and placed in individual feed bunks equipped with an electronic feeding system (American Calan; Northwood, NH) that only allowed access of one specified heifer to consume the feed. From day 106 to parturition, heifers were fed a total mixed ration (TMR) in an electronic feeding system (Insentec Roughage Intake Control Systems; Hokofarm B.V., Marknesse, The Netherlands), with the VTM heifers continuing to receive the loose of vitamin and mineral supplement incorporated into the TMR. The heifers were weighed every two weeks, and feed deliveries were modified as necessary to meet the goal gains of 0.45 kg·heifer^−1^·d^−1^.

Heifers were bred via AI using female-sexed semen from a single sire and evaluated for pregnancy diagnosis via transrectal ultrasound at day 35 after the AI was performed and on day 65 to determine fetal sex [32]. Only heifers confirmed pregnant with female fetuses were used. The treatments were maintained from breeding to parturition. At calving, dams were monitored to collect the placentas upon expulsion within 24 h. After collection, the placenta was spread out on a clean table to localize the largest COT nearest to the umbilical cord, which was manually dissected for further analysis.

### 2.3. Tissue Collection and Analysis

In Exp. 1, whole placentomes were collected and fixed in neutral buffered formalin (NBF). The largest placentome closest to the umbilical cord was collected and manually dissected; one part was fixed in NBF and the other snap-frozen in liquid nitrogen for rapid preservation and stored at ultra-low temperature (−80 °C) for subsequent analysis. In Exp. 2, the largest COT located nearest to the umbilical cord was collected and fixed in NBF. In both experiments, the collected tissue was fixed for a duration of 24 h in NBF and then transferred into 70% ethanol. Fixed blocks were embedded in paraffin via a tissue processor (Leica Biosystems Inc., Buffalo Grove, IL, USA). Slides were cut on a microtome at 5 µm thickness for 3-D analysis of vascularity.

### 2.4. Immunohistochemistry

Tissue sections fixed in NBF were used for immunohistochemistry analysis. As previously described [33], rabbit anti-CD34 and rabbit anti-CD31(Abcam, Cambridge, MA, USA) were used as markers for endothelial cells to quantify vascularity, and DAPI was used for background nuclear staining. Additionally, for the parturition study, fluorescein Griffonia Simplifolia lectin I (BS1 lectin; Vector Laboratories, Burlingame, CA, USA) was used as a marker of fetal tissue. Sections were deparaffinized in xylene (3 times × 5 min each), 100% alcohol (2 times × 5 min each), 95% alcohol (1 time × 5 min), and 70% alcohol (1 time × 5 min), and, finally, in distilled water (1 time × 5 min) for rehydration. Epitope retrieval was performed in Na-citrate buffer for 30 min at 121 °C. Antigen blocking was performed in 5% normal goat serum (Vector Laboratories, Burlingame, CA, USA) at room temperature for 1 h.

**Figure 1:**
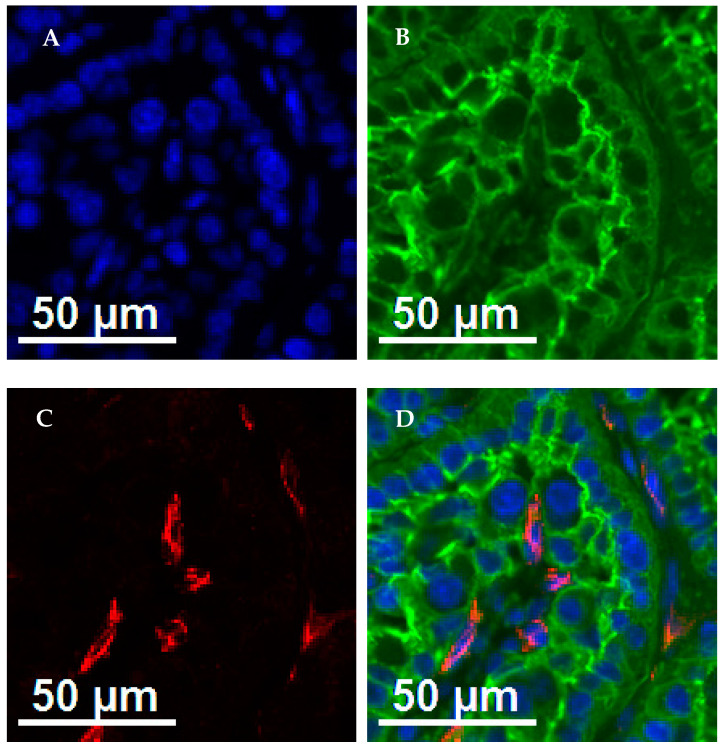
Immunohistochemistry of placental (maternal and fetal) samples from beef heifers receiving or not vitamin and mineral supplementation and fed to achieve different rates of body weight gain (low gain (LG) or moderate gain (MG)) during early gestation (Exp. 1). (**A**) Background nuclear staining with DAPI; (**B**) fetal placenta stained for BS1 lectin; (**C**) endothelial cells of blood vessels stained with CD-31 and CD-34 antibodies; and (**D**) DAPI (blue) + BS1 lectin (green) + CD-31 + CD-34 (red). Size bar indicates 50 μm.

In Exp. 1, primary CD-34 and primary CD-31 monoclonal antibodies (Abcam) were diluted at 1:500 and 1:50, respectively, in 1% bovine serum albumin (BSA; Avantor, Visalia, CA, USA) and incubated at room temperature for 1 h. After incubation with the primary antibodies, the secondary CF-633 goat anti-rabbit antibody (1:250 in 1% BSA, Biotium, Fremont, CA, USA) was incubated with tissue sections for 1 h at room temperature. BS1 Lectin (1:200 in 1% BSA) was incubated with tissue sections for 30 min. Finally, nuclear staining for the background was performed with DAPI at room temperature for 5 min (Figure 1).

In Exp. 2, the primary CD-34 monoclonal antibody (1:500 in 1% BSA; Abcam) was incubated overnight at 4 °C. The next day, the primary CD-31 monoclonal antibody (1:50 in 1% BSA; Abcam) was incubated with tissue sections for 1 h at room temperature. Secondary CF-633 goat anti-rabbit antibody (1:250 in 1% BSA; Biotium, Fremont, CA, USA) was incubated at room temperature for 1 h with tissue sections. Lastly, nuclear staining for the background was applied with DAPI for 5 min at room temperature.

### 2.5. Image Analysis

Images were captured with a Zeiss AxioImager M2 epifluorescence microscope using a 20× 0.8 NA objective and an AxioCam HRm camera. Images were further processed and saved with Zeiss AxioVision Rev. 4.8.1 image analysis software (Carl Zeiss, Thornwood, NY, USA). Images were analyzed using ImagePro-Premiere software (Ver.9.0.1 Media Cybernetics, Inc., Silver Spring, MD, USA). Briefly, in both studies, the areas of interest for COT (fetal placenta) were carefully outlined and analyzed for the percentage of blood vessels within the tissue area, using the standardized segmentation values that allowed objective comparisons of the samples.

### 2.6. Gene Expression

Taking advantage of transcriptomic data from the same study (Exp. 1), we retrieved the expression values of eight angiogenic/vasoactive factor genes (*ANGPT*-1, *ANGPT*-2, *eNOS*2, *eNOS*3, *FLT*1, *KDR*, *TEK*, and *VEGFA*). The RNA-Seq data from COT tissues from 31 heifers were previously used to identify differentially expressed genes in response to different maternal planes of nutrition [34]. Experimental and analytical procedures for quality control, sequencing, alignment, and bioinformatics are provided elsewhere [34]. Briefly, strand-specific RNA libraries were prepared and sequenced on the Illumina^®^ platform to generate paired-end 150 bp reads. Read mapping and counting were performed using STAR aligner [35] and the *Bos taurus* reference genome [36]. Read counts from STAR were used as input for data quality control using the R-package DESeq2 v.1.22.1 [37]. Genes with no expression were filtered out. Next, the gene count data were normalized through the VST function from DESeq2. Targeted genes were retrieved, and normalized expression values were used to create boxplots using the ggplot2 v.3.4.2 R-package [38].

### 2.7. Statistical Analysis

In both experiments, for the image analysis, three different images from each heifer were obtained, and their means were used for the statistical analysis. Mean values of the vascular area density (percentage of tissue area represented by CD-31 and CD-34 staining) were calculated for each animal. Data were analyzed using the GLM procedure of SAS (SAS version 9.4; SAS Inst. Inc., Cary, NY, USA). In Exp. 1, data were analyzed as a 2 × 2 factorial, and model terms included VTM (VTM or NoVTM), GAIN (LG or MG), and their interaction. In Exp. 2, the model term included treatment (VTM or NoVTM). For all analyses, the heifer was the experimental unit. A *p*-value < 0.1 was considered significant.

## 3. Results

### 3.1. Placental Vascular Area Density

**Figure 2:**
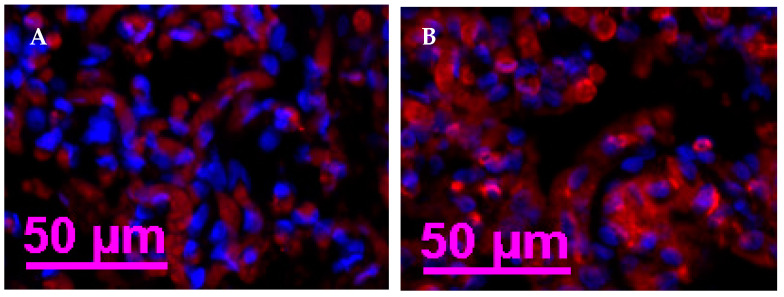
Immunohistochemistry of COT from beef heifers receiving or not vitamin–mineral supplementation from breeding to parturition (Exp. 2). (**A**) CON = control (no supplement) group; (**B**) VTM (supplemented) group. Blue indicates background nuclear staining with DAPI and red indicates endothelial cells of blood vessels stained with CD-31 + CD-34. Size bar indicates 50 μm.

In Exp. 1, COT vascularity was not affected by the interaction of VTM and GAIN (*p* = 0.66) and the main effects of VTM (*p* = 0.50) and GAIN (*p* = 0.54). However, in Exp. 2, COT vascularity was greater (*p* = 0.07) in the VTM group compared to the CON group (Table 2, Figure 2).

**Figure 3:**
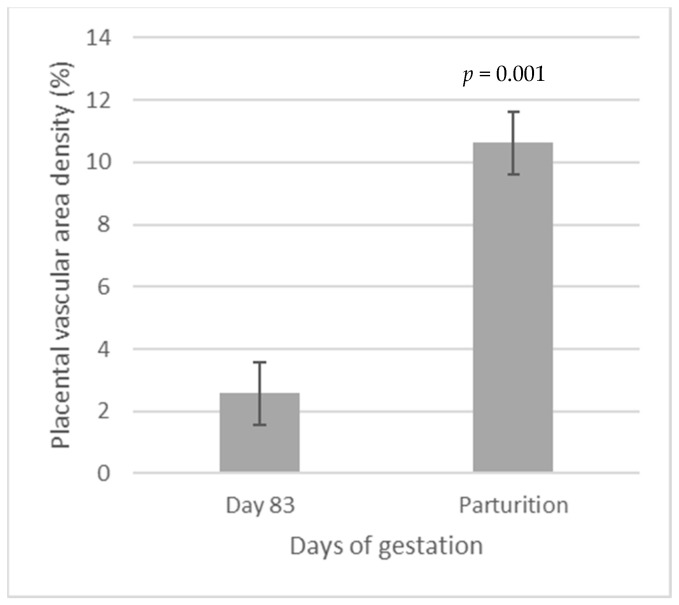
Comparison of fetal placental (COT) vascular area density between day 83 (2.57%) and at parturition (10.62%); a 4-fold increase was observed from beef heifers receiving or not vitamin–mineral supplementation from breeding to early gestation (day 83) vs. throughout gestation. This measurement was obtained through immunohistochemistry, utilizing CD-34 and CD-31 as markers for endothelial cells within blood vessels, followed by image analysis.

When we compared placental vascularity between day 83 and at parturition, we observed that placentas collected at parturition had greater (I = 0.001, Figure 3) vascularity.

### 3.2. RNA Expression of Placental Angiogenic/Vasoactive Factors and Their Receptors on Day 83 of Gestation

No differences (*p* ≥ 0.25) were observed among treatments for gene expression of any of the angiogenic/vasoactive factors or their receptors evaluated in COT (Table 3).

## 4. Discussion

Placental angiogenesis and normal development of blood vessels play a crucial role in establishing a correct placental function and successful pregnancy [1,5,7]. In this study, we hypothesized that dietary vitamin and mineral supplementation and/or different rates of body weight gain through pregnancy would enhance placental vascular development and improve fetoplacental function in crossbred Angus heifers. Several studies have demonstrated that maternal nutrition during gestation significantly impacts placental development, fetal programming, and offspring’s subsequent health [39].

Kanjanaruch et al. [39] showed an effect on placental vascularity during early pregnancy in response to dietary restriction and one-carbon metabolite (methionine, choline, folate, and vitamin B12) supplementation. McLean et al. [40] described differences in placental vascularity and mRNA expression of angiogenic factors in heifers under a nutrient-restriction diet during the first 50 days of gestation. However, our results indicate that maternal supplementation with vitamins, minerals, and/or protein/energy from pre-breeding to day 83 did not significantly affect placental vascular development. Interestingly, the supplementation with vitamins and minerals throughout the entire gestation period appeared to enhance placental vascularity at parturition. This suggests an effect emerging in a later stage, possibly attributed to the fact that most placental development occurs during mid-gestation rather than early gestation [33].

The complexity of the study on micronutrient supplementation relies upon the complicated relationship between minerals and their mechanisms, making it difficult to identify the specific mechanism causing the observed outcomes. For Exp. 2, the trace mineral concentration at day 84 and 180 of gestation of the dams and the placental COT at parturition is described in Hurlbert et al. [31]. They reported a greater concentration of hepatic Se in VTM heifers compared to CON heifers at day 84 and 180 of gestation. However, there was a decrease in the hepatic concentration of Se in the VTM heifers and a decrease in Cu in both groups (VTM and CON) from day 84 to parturition in the dam. Placental trace mineral concentration indicated a greater Se concentration in VTM heifers than CON heifers. These results suggest a potential placental vascular enhancement in response to greater levels of Se. Previous studies of maternal supplementation with Se during pregnancy have shown an effect on vascular development [24]. Vonnahme et al. [41] described a vascularity increase in mammary glands of post-partum ewes in response to Se supplementation; however, no differences were found in the mRNA expression of VEGF. In addition, Lekatz et al. [42] showed an effect in the mRNA levels of VEGF, VEGFR-1, VEGFR-2, and NO synthase in the COT of ewes supplemented with Se and different nutrition plans through gestation.

Previous findings from Diniz et al. [27,34] demonstrated that VTM supplementation led to distinct expression patterns in placental genes involved with energy metabolism and nutrient transport from the same heifers used in Exp. 1. However, the angiogenic/vasoactive factor-coding genes were not differentially expressed following nutritional supplementation up to day 83. Furthermore, the data about the transcript abundance of COT, including nutrient transporters and angiogenic factors from Exp. 2, are critical to gaining a better understanding of the mechanisms underlying the possible relation between vitamin and mineral supplementation and placental vascular development. For placental angiogenesis to occur, creating new blood vessels requires the existing trophoblastic microvessels to undergo endothelial cell proliferation, migration, and differentiation [43]. When maternal dietary intake in pregnant adolescent sheep was altered, there was no effect on placental vascular area density at day 81 of gestation (approximately equivalent to day 154 of gestation in cattle). However, the authors reported that the expression of *VEGF*, *ANGPT*1, *ANGPT*2, *eNOS*3, and one of their receptors (*FLT*1) was reduced, and was reflected by a reduced placental vascularity in late pregnancy [44]. In addition, these studies found a significant negative correlation between maternal body weight gain and placental mRNA expression for several angiogenic factors, suggesting that maternal nutrient intake may regulate placental angiogenic factor expression [44]. Even so, the achieved differences in maternal body weight as we targeted in our model in Exp. 1 (0.28 kg/d in LG group vs. 0.79 kg/d in MG group) were not sufficient to cause differences in COT mRNA expression of angiogenic factors or vascular area density during the first trimester of gestation. Nevertheless, it is known that up until mid-gestation, the fetal COT appears to have relatively poor vascularization, with greater levels of *VEGF* expression being found at this stage. At this point, fetal COT undergoes dramatic cellular proliferation and tissue remodeling, resulting in increased vascularization [33]. This agrees with our finding of greater COT vascularity in Exp. 2 compared with Exp. 1. However, it is important to note that mid-gestation represents a later stage within the gestational timeline from that examined in our current study (day 83 vs. day 140 for mid-gestation). Unfortunately, we were unable to quantify angiogenic/vasoactive factor gene expression in the delivered placentas at parturition.

Additionally, Hurlbert et al. [31] recorded and reported calf weight at birth, weaning, and post-weaning time points. No differences were found between treatments on calf weight at birth. Calves receiving *in utero* vitamin and mineral supplementation throughout gestation had a heavier weight at weaning and post-weaning than the CON calves. These results suggest that dietary vitamin and mineral supplementation from pre-breeding and throughout gestation could benefit placental vascular development, enhancing placental function that manifests at a later post-natal stage. Additional mechanisms that might contribute to improving placental function in response to vitamin and mineral supplementation include the modulation of hormonal pathways and the impact on the microbiome [45,46,47,48]. Estrogens, identified as key players in angiogenic factor expression during placental development, function crucially in developmental processes responsible for fetal growth and healthy offspring [45,46]. Furthermore, the maternal microbiome has been demonstrated to enhance placental growth and vascular development in mice [47], additionally Amat et al. [48] conducted the characterization of the microbiota of bovine fetuses, amniotic and allantoic fluid, and COT from early gestation (Exp.1), resulting in a modified fetal microbiome; however, no effect on COT microbiome was found. Therefore, the effects of vitamin and mineral supplementation throughout the complete gestational period on maternal steroid hormones and microbiome and its relationship with placental development in large animal species remain unexplored and demand further investigation.

## 5. Conclusions

Our results confirmed a complex relationship between maternal nutrition, placental function, and offspring development. We showed increased placental vascularity from beef heifers receiving vitamin and mineral supplementation throughout gestation, potentially in response to a greater Se level in VTM heifers. This finding suggests that vitamin and mineral supplementation, particularly throughout late gestation, may positively influence placental development, which manifests in postnatal benefits, such as enhanced calf weights at weaning and post-weaning. While we just evaluated angiogenic factor gene expression at day 83 of gestation, it is apparent that the full spectrum of effects may extend into later gestational stages. Furthermore, the timing of supplementation and its specific impact on angiogenic factors in the placenta may vary.

## Figures and Tables

**Table 1:** Nutrient composition of total mixed ration and supplements provided during the first trimester of gestation [29].

Composition	TMR ^1^	NoVTM ^2^	VTM ^3^	RG ^4^
Dry matter, %	53.0	86.6	89.6	87.7
Ash, % DM	11.5	5.3	25.1	2.4
Crude protein, % DM	9.9	15.6	14.8	17.5
Neutral detergent fiber, % DM	65.9	41.9	27.6	19.4
Ether extract, % DM	1.5	-	-	9.1
Non-fiber carbohydrates, % DM	11.1	37.2	32.5	51.6
Mineral Content				
Calcium, g/kg DM	5.74	2.47	50.62	0.30
Phosphorus, g/kg DM	2.05	8.94	22.82	4.59
Sodium, g/kg DM	0.26	0.12	19.44	0.24
Magnesium, g/kg DM	2.83	4.47	5.20	1.96
Potassium, g/kg DM	15.81	14.22	13.15	6.05
Sulfur, g/kg DM	2.25	2.41	4.84	2.57
Manganese, mg/kg DM	121.2	103.9	953.4	26.0
Cobalt, mg/kg DM	0.36	0.14	3.38	0.05
Copper, mg/kg DM	4.8	13.7	285.8	3.6
Selenium, mg/kg DM	0.3	0.4	7.0	0.3
Zinc, mg/kg DM	28.4	130.2	1051.8	35.0

^1^ Total Mixed Ration: Proportion of ingredients: prairie grass hay (55%), corn silage (38%) and dried distillers’ grains plus solubles (7%); this group served as the Control group. ^2^ NoVTM: No vitamin-mineral supplement was a pelleted product fed at 0.44 kg/heifer daily with no added vitamin and mineral supplement. ^3^ VTM: Vitamin mineral supplement was a pelleted product fed at 0.44 kg/heifer daily (consisting of 113 g of a vitamin and mineral supplement [Purina Wind & Rain Storm All-Season 7.5 Complete, Land O’Lakes Inc., Arden Hills, MN, USA] and 337 g of a carrier). ^4^ Starch-based protein/energy: An energy/protein supplement formulated with a blend of ground corn, dried distillers’ grains plus solubles, wheat midds, fish oil, and urea; targeting gain of 0.79 kg/d for moderate-gain and 0.28 kg/d for low-gain heifers.

**Table 2:** Vascular area density of COT (fetal placenta) from beef heifers receiving or not vitamin–mineral supplementation and fed to achieve different rates of body weight gain (low gain (LG) or moderate gain (MG)) during early gestation (Exp. 1) (day 83 of gestation) and from beef heifers receiving or not vitamin–mineral supplementation from breeding to parturition (Exp. 2).

	Experiment 1	
Treatments *	Vascular Area Density (%)	SEM and *p*-Values
NoVTM	2.66 ^A^	0.19
VTM	2.45 ^A^	0.19
Main effect of VTM		*p* = 0.50
LG	2.49 ^A^	0.19
MG	2.65 ^A^	0.19
Main effect of GAIN		*p* = 0.54
NoVTM-LG	2.52 ^A^	0.27
NoVTM-MG	2.80 ^A^	0.27
VTM-LG	2.46 ^A^	0.27
VTM-MG	2.50 ^A^	0.27
VTM × GAIN		*p* = 0.66
	Experiment 2	
CON	8.65 ^A^	1.39
VTM	12.09 ^B^	1.20
Main effect of VTM		*p* = 0.07

* NoVTM = no vitamin–mineral supplement; VTM = vitamin + mineral supplement; LG = low rate of gain; MG = moderate rate of gain; CON = control (no supplement). ^AB^ indicates significant differences. Means lacking a common superscript differ at a significance level of *p* < 0.1.

**Table 3:** Gene expression of angiogenic factors of fetal placental tissue (COT) from beef heifers receiving or not vitamins and minerals supplementation from breeding to early gestation (day 83, Exp. 1).

Gene *	Treatment Mean					
	VTM-LG	VTM-MG	NoVTM-LG	NoVTM-MG	SEM	*p*-Value
*VEGF-A*	11.37 ^A^	11.59 ^A^	11.57 ^A^	11.59 ^A^	0.17	0.61
*FLT1*	9.35 ^A^	9.54 ^A^	9.47 ^A^	9.54 ^A^	0.14	0.65
*KDR*	9.94 ^A^	10.11 ^A^	10.03 ^A^	10.11 ^A^	0.18	0.81
*ANGPT-1*	4.77 ^A^	4.64 ^A^	4.82 ^A^	4.65 ^A^	0.10	0.53
*ANGPT-2*	6.97 ^A^	7.08 ^A^	6.99 ^A^	7.08 ^A^	0.10	0.80
*TEK*	9.19 ^A^	9.18 ^A^	9.06 ^A^	9.18 ^A^	0.12	0.47
*eNOS2*	8.75 ^A^	8.68 ^A^	8.70 ^A^	8.68 ^A^	0.23	0.25
*eNOS3*	7.51 ^A^	7.68 ^A^	7.45 ^A^	7.68 ^A^	0.16	0.43

* *VEGF-A* = vascular endothelial growth factor A; *FLT*1 = vascular endothelial growth factor receptor 1; *KDR* = tyrosine kinase growth factor receptor; *ANGPT*-1 = angiopoietin 1; *ANGPT*-2 = angiopoietin 2; *TEK* = receptor tyrosine kinase; *eNOS2* = endothelial nitric oxide synthase 2; *eNOS*3 = endothelial nitric oxide synthase 3. ). ^A^ indicates significant differences. Means lacking a common superscript differ at a significance level of *p* < 0.1.

## Data Availability

Data available on request.
